# Topical treatments for Kaposi sarcoma: A systematic review

**DOI:** 10.1002/ski2.107

**Published:** 2022-04-08

**Authors:** Kyaw Zin Htet, Michael Andrew Waul, Kieron Seymour Leslie

**Affiliations:** ^1^ Tulane University School of Medicine New Orleans Louisiana USA; ^2^ Department of Dermatology University of California San Francisco California USA

## Abstract

**Background:**

While treatment options exist for solitary or disseminated Kaposi sarcoma (KS) disease, there are currently no standardized clinical treatment guidelines for cutaneous KS.

**Objective:**

This systematic review seeks to identify safe and effective topical treatments for cutaneous KS lesions.

**Methods:**

We conducted a systematic review using peer‐reviewed articles from January 1970 to September 2021 published in the PubMed/MEDLINE and EMBASE databases.

**Results:**

From the initial search that yielded 590 studies, 34 met the inclusion criteria and were selected. Of the 34 studies, seven were clinical trials, 26 were case reports/series and one was a multicentre study. A total of 634 patients were included in our review. The three most common topical treatments used for cutaneous KS were imiquimod, alitretinoin and timolol. Topical alitretinoin was used in three case reports and three clinical trials. Topical imiquimod was used in eight case reports, one prospective phase II cohort study and one comparative single‐blinded non‐controlled clinical study. Topical timolol was used in nine case reports/series. Our review also identified reports of less widely used topical treatments for cutaneous KS. These include topical diphencyprone (DPCP), all‐*trans*‐retinoic‐acid, rapamycin and bleomycin‐dimethylsulfoxide (BLM‐DMSO) which achieved variable response rates but have not been widely studied.

**Conclusion:**

Topical alitretinoin, imiquimod and timolol demonstrated positive responses for cutaneous KS and the treatments were well tolerated. These three topical treatment modalities could be considered by clinicians when treating cutaneous KS.

## Graphical abstract

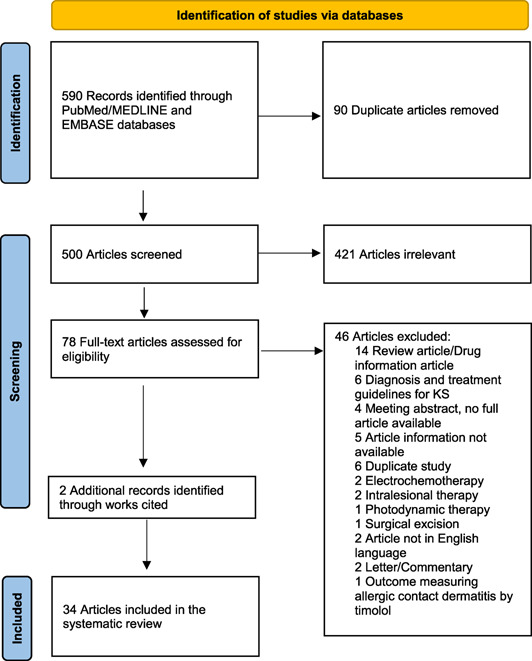

1

1 **What is already known about this topic?**

- Kaposi sarcoma (KS) is an angioproliferative tumour that is associated with human herpesvirus‐8 (HHV‐8). The treatment modalities for KS depend on the extent and involvement of the lesions, ranging from clinical observation to systemic chemotherapeutic agents. However, there are currently no standardized clinical treatment guidelines for cutaneous KS.

2 **What does this study add?**

- Our systematic review identified that the three most common topical treatments for cutaneous KS are alitretinoin, imiquimod and timolol. All three treatments for cutaneous KS are well tolerated and demonstrated positive responses.

## 2 INTRODUCTION

Kaposi sarcoma (KS) is an angioproliferative tumour that is associated with human herpesvirus‐8 (HHV‐8). There are four known clinical categories of KS: classic, endemic, iatrogenic and epidemic or AIDS‐related KS.^1^ KS typically presents with violaceous patches, plaques and/or nodules on the skin or mucocutaneous surfaces. In some cases, the disease can manifest as a solitary lesion in a limited area, or as multiple lesions in a diffuse pattern throughout the body. The treatment of KS depends on the clinical category, the extent of lesion involvement and the pre‐existing medical conditions of the patient.^2^ Therapeutic options for management of KS range from clinical observation for indolent solitary lesions, initiation or optimization of antiretroviral therapy for AIDS‐related KS, to systemic chemotherapeutic agents for disseminated disease.^3^ For patients with cutaneous KS, recent clinical guidelines have recommended radiotherapy, surgical excision, cryosurgery, laser therapy, and intralesional agents as treatment modalities.^2^, ^4^ Although these treatments are effective in most cases, they may not be feasible for all patients; their use is dependent on patient comorbidities, as well as the anatomical locations and extent of KS lesions. Topical treatment may be more appropriate for some cases of cutaneous KS, as they potentially allow higher drug levels at the tumour site, lower degrees of pain and scarring, and have better safety profiles. The objective of this systematic review is to characterize all the topical treatment modalities for cutaneous KS and evaluate the clinical efficacy and safety of each topical treatment.

## 3 METHODS

This systematic review follows the Preferred Reporting Items for Systematic Reviews and Meta‐Analysis (PRISMA) guidelines.^5^

### 3.1 Eligibility criteria

We reviewed randomized controlled trials, retrospective cohort studies, prospective cohort studies, case series and case reports that utilized topical agents for treatment of cutaneous KS. The following inclusion criteria were used: studies that used topical treatments only, or studies that compared treatment groups between a topical agent and another local or systemic agent. Only articles written in English language were included. Studies were excluded if a non‐topical treatment was used for cutaneous KS. Studies were also excluded if other systemic or local treatments were used in conjunction with topical treatments, or if they did not use clinical response as an outcome measure. Local treatments were defined as non‐topical agents including radiotherapy, surgical excision, cryosurgery, laser, and intralesional agents. Additionally, review articles, study protocols, letters‐commentaries‐editorials, and guidelines‐recommendations were excluded.

### 3.2 Information sources and search strategy

A search of two databases, including PubMed/MEDLINE and EMBASE, from January 1970 to September 2021 was conducted. The following Medical Subject Headings and keywords were used appropriate to each database: ‘Kaposi sarcoma’ or ‘Kaposi's sarcoma’ and ‘topical treatment’ or ‘topical administration’ or ‘topicals’ or ‘topical therapeutics’. Subsequent review of relevant article bibliographies was conducted to identify any additional studies. Full search strategy is available in the supplementary document.

### 3.3 Selection process

All articles retrieved through database search were imported into Covidence software, where duplicates were automatically removed. Two reviewers (Kyaw Zin Htet and Michael A. Waul) independently assessed the eligibility of each article through an initial title and abstract review. Reviewers were blinded to each other's assessments. Irrelevant articles were excluded. Following title and abstract review, the full text of remaining articles was reviewed for eligibility. Full texts were assessed for eligibility independently and in duplicate. Any full text articles that did not meet eligibility criteria were excluded. Disagreements were resolved by a third author (Kieron S. Leslie) as a tie breaker.

## 4 DATA COLLECTION PROCESS

All included studies were extracted using a pre‐specified extraction spread sheet by two independent reviewers (Kyaw Zin Htet and Michael A. Waul). The data from the search were extracted and the following information when available was ascertained: patient age and sex, number of cases, HIV status, type of KS, known presence of extracutaneous involvement, drug response, drug regimen, timeline of sustained response and drug‐related adverse side effects. The primary summary measure was the clinical outcome of topical treatment for cutaneous KS.

### 4.1 Study risk of bias assessment

The risk of bias was assessed depending on study design, utilizing the Newcastle‐Ottawa Quality Assessment Scale (NOS) for case control studies, cohort studies and randomized controlled trials.^6^ Case reports and case series were assessed using a modified NOS.^7^ This was performed independently by two independent reviewers (Kyaw Zin Htet and Michael A. Waul) and any discrepancies were reviewed by a third reviewer (Kieron S. Leslie). The NOS tool includes eight points that evaluate the selection, comparability and exposure described in the studies. Points are awarded based on each evaluation criterion and tallied to form a final quality score. The minimum score is 0 and maximum score is 9. For purposes of this review, high quality was defined by scores ranging from 7 to 9, moderate quality was defined by scores ranging from 4 to 6, and poor quality was defined by scores less than 4. The breakdown of NOS scores for each included article is illustrated in Tables S5 and S6 of the supplementary document.

### 4.2 Synthesis methods

Each included individual article was rated using the modified Oxford Centre for evidence‐based medicine rating scale.^8^ Extracted outcome data, NOS score and ratings for each article are summarized in tabulated formats.

## 5 RESULTS

**FIGURE 1:**
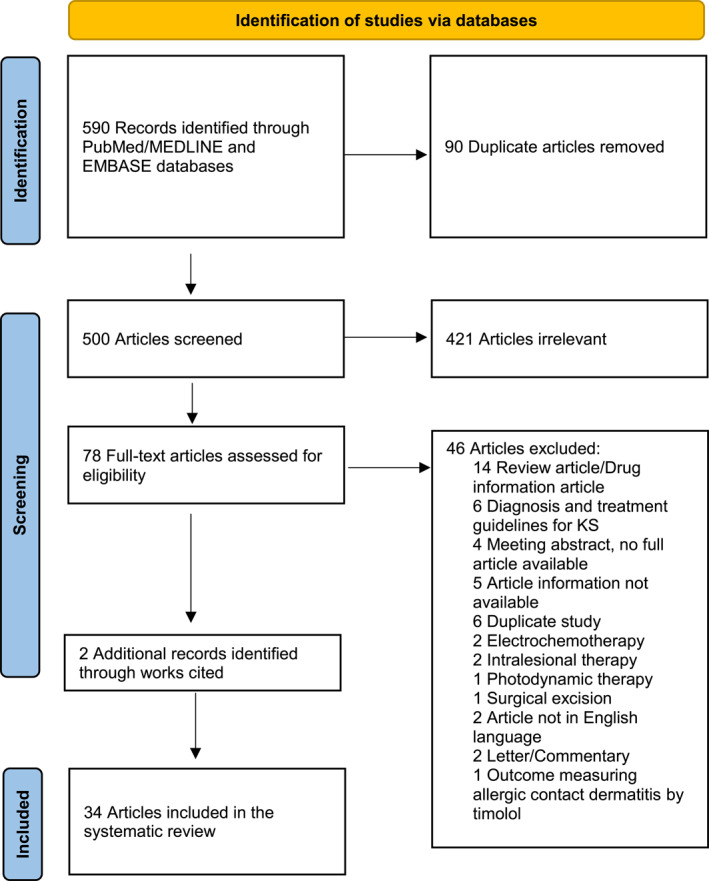
PRISMA flow diagram

The search results yielded a total of 589 studies that were published from January 1970 to September 2021. After duplicate studies were removed, remaining studies were screened based on title, abstract and full text to assess for eligibility. An additional two studies were identified through bibliography review and included, to produce a total of 34 studies eligible for inclusion. Of these studies, 26 were case reports or series and 8 were randomized clinical trials (RCTs). The selection process is shown in the format of a PRISMA diagram in Figure 1.

### 5.1 Demographics

In the 34 studies included in this systematic review, the majority of patients were male 609/633 (96.2%), with only 24/633 female (3.8%). Two studies did not have gender specifications.^9^, ^10^ Within our included studies, there were 544 cases of AIDS‐related KS, 120 cases of classic KS, 6 cases of iatrogenic KS, and 1 case of endemic KS. One study did not report any KS classifications,^9^ and another grouped 17 cases of endemic and AIDS‐related KS together.^11^ The median age of patients in the included studies was 64.5 years with a range of 25–94 years. For patients with classic KS, the median age was 74 years, for AIDS‐related KS 38.5 years, iatrogenic KS 65 years and endemic KS 42 years. For detailed summary of demographics see Tables 1, 2, 3, 4.

**TABLE 1:** Summary of demographics, clinical characteristics and outcomes of topical alitretinoin for cutaneous Kaposi sarcoma (KS)

Source, y	Study design	Age, median (range)	Male/female no.	HIV status	Type of KS	Known presence of extracutaneous diseases	Response	Regimen	Sustained response	LOE^a^/NOS score^b^
Bodsworth et al.,^12^ 2001	Randomized phase III double‐blinded study (*n* = 62)	38	134/0	All HIV+	62 AIDS‐KS	None	(*n* = 62) 1 CR, 22 PR, 27 SD, 12 PD	0.1% topical alitretinoin gel two times per for 12 weeks or until confirmation of disease progression	Did not follow‐up	1/8
Duvic et al.,^13^ 2000	Randomized phase I and II open‐label controlled study (*n* = 115)	38 (25–64)	115/0	All HIV+	115 AIDS‐KS	1 case—visceral KS	(*n* = 115) 3 CR, 28 unspecified responses	0.05% or 0.1% alitretinoin gel two times per day for the 2 weeks with subsequent dose increase up to maximum of 0.1% four times per day	Did not follow‐up	2/7
González de Arriba et al.,^14^ 2007	Case report (*n* = 1)	83	0/1	Negative	IKS	None	CR	0.1% topical alitretinoin gel two times per day but reduced to only one application every 48–72 h for 5 months	Did not follow‐up	5/6
Morganroth,^15^ 2002	Case report (*n* = 1)	83	0/1	Not tested	CKS	None	CR	0.1% topical alitretinoin gel two times per day for 1.5 years	No recurrence of KS in the treated sites after 18 months follow‐up	5/7
Rongioletti et al.,^16^ 2006	Case report (*n* = 1)	75	1/0	Negative	CKS	Bilateral leg and pedal oedema	NR	0.1% topical alitretinoin gel two times per day for 3 months	Progression of the lesions after 3–5 months follow‐up	5/7
Walmsley et al.,^17^ 1999	Randomized double‐blinded multi‐centre clinical trial (*n* = 134)	39 (26–71)	266/2	All HIV+	134 AIDS‐KS	32 cases of visceral KS	(*n* = 134)—1 CR, 46 PR, 67 SD, 20 PD	0.1% topical alitretinoin gel three times per day for the first 2 weeks of study, followed by four times per day for the remainder of the study	Did not follow‐up	1/8

Abbreviations: AIDS‐KS, AIDS‐related KS; CKS, Classical KS; CR, Complete Response; IKS, Iatrogenic KS; LOE, level of evidence; NOS, Newcastle‐Ottawa Quality Assessment Scale; NR, no response; PD, progressive disease; PR, partial response; SD, stable disease.

^a^ Level of evidence rated using modified Oxford Centre for evidence‐based medicine.

^b^ The risk of bias for included studies assessed using Newcastle‐Ottawa Quality Assessment Scale.

**TABLE 2:** Summary of demographics, clinical characteristics and outcomes of topical imiquimod for cutaneous Kaposi sarcoma (KS)

Source, y	Study design	Age, median (range)	Male/female no.	HIV status	Type of KS	Known presence of extracutaneous diseases	Response	Regimen	Sustained response	LOE^a^/NOS score^b^
Babel et al.,^18^ 2007	Case report (*n* = 1)	65	1/0	Not tested	IKS	None	CR	5% imiquimod cream for 13 weeks	No recurrence of KS after 15 months follow‐up	5/7
Benomar et al.,^19^ 2009	Case report (*n* = 1)	57	1/0	Negative	CKS	None	CR	5% imiquimod cream three times a week under occlusion for 10–12 h for 20 weeks	No recurrence of KS after 6 months follow‐up	5/5
Célestin Schartz et al.,^11^ 2008	Prospective phase II cohort study (*n* = 17)	68.5 (60–76)	14/3	All negative	17 endemic KS/CKS	None	2 CR, 6 PR, 6 PD	5% imiquimod cream under occlusion three times a week for 24 weeks	Did not follow‐up	2/4
Fairley et al.,^20^ 2012	Case report (*n* = 1)	43	1/0	Negative	CKS	None	CR	5% imiquimod cream for 6 weeks	No recurrence of KS after 15 months follow‐up	5/6
Goiriz et al.,^21^ 2008	Case report (*n* = 1)	87	1/0	Negative	CKS	None	CR	Topical imiquimod was initially applied once daily and left overnight under occlusion for 6 months with subsequent tapering of frequency	No recurrence of KS after 12 months follow‐up	5/5
Rosen,^22^ 2006	Case report (*n* = 1)	41	1/0	HIV+	AIDS‐KS	None	PR	5% imiquimod cream daily for 4 months	No recurrence of KS after 6 months follow‐up	5/7
Bernardini et al.,^23^ 2010	Case report (*n* = 1)	77	1/0	Negative	CKS	None	CR	5% imiquimod cream 3 times a week under occlusion overnight for at least 8 h for 3 months	No recurrence of KS after 12 months follow‐up	5/6
Gündüz et al.,^24^ 2012	Case report (*n* = 1)	74	1/0	Negative	CKS	None	CR	5% imiquimod cream three times a week under occlusion for 3 months	No recurrence of KS after 6 months follow‐up	5/5
Prinz Vavricka et al.,^25^ 2011	Case report (*n* = 2)	61.5 (51–72)	1/1	All negative	2 IKS	None	1 CR, 1 PR	1 case—5% imiquimod cream daily for 8 h under occlusion for 19 months, 1 case—5% imiquimod cream without occlusion daily for 4 months	No recurrence of KS after 12 and 96 months follow‐up	5/8
Odyakmaz Demirsoy et al.,^26^ 2019	Comparative single‐blinded noncontrolled clinical study (*n* = 8, 29 lesions total)	65.1 (37–82)	2/6	All negative	50 CKS	None	(*n* = 29) 11 CR, 8 PR, 4 FR, 3 NR, 3 lost for follow up	5% imiquimod cream overnight without occlusion for at least 8 h three times a week for 12 weeks	Did not follow‐up	2/4

Abbreviations: AIDS‐KS, AIDS‐related KS; CKS, Classical KS; CR, Complete Response; IKS, Iatrogenic KS; LOE, level of evidence; NOS, Newcastle‐Ottawa Quality Assessment Scale; NR, no response; PD, progressive disease; PR, partial response; SD, stable disease.

^a^ Level of evidence rated using modified Oxford Centre for evidence‐based medicine.

^b^ The risk of bias for included studies assessed using Newcastle‐Ottawa Quality Assessment Scale.

**TABLE 3:** Summary of demographics, clinical characteristics and outcomes of topical timolol for cutaneous Kaposi sarcoma (KS)

Source, y	Study design	Age, median (range)	Male/female no.	HIV status	Type of KS	Known presence of extracutaneous diseases	Response	Regimen	Sustained response	LOE^a^/NOS score^b^
Abdelmaksoud et al.,^27^ 2017	Case series (*n* = 4)	58.5 (45–70)	3/1	One case of HIV+	3 CKS, 1 AIDS‐KS	None	3 CR, 1 PR	0.1% topical timolol gel two times per day until resolution (4, 5, 5 and 6 weeks)	No recurrence of KS after 4, 6, 9 and 10 months follow‐up	4/7
Alcántara‐Reifs et al.,^28^ 2016	Case report (*n* = 2)	86 (83 –89)	2/0	All negative	2 CKS	None	All CR	0.5% topical timolol gel for 12 and 18 weeks	No recurrence of KS after 4 and 5 months of follow‐up	5/5
Chap et al.,^29^ 2017	Case report (*n* = 1)	61	1/0	Negative	IKS	Bilateral inguinal lymph node involvement	PR	0.5% topical timolol gel for 17 weeks	No recurrence of KS after 4 months follow‐up	5/6
Deutsch et al.,^30^ 2018	Case report (*n* = 1)	42	1/0	Negative	1 Endemic KS	None	CR	Did not specify	Unclear timeline of follow‐up	5/3
Espadafor‐López et al.,^31^ 2020	Case report (*n* = 1)	70	0/1	Negative	IKS	None	CR	0.5% topical timolol gel two times per day for 16 weeks	No recurrence of KS after 6 months follow‐up	5/6
Gupta et al.,^32^ 2019	Case report (*n* = 1)	55	0/1	Negative	CKS	None	NR	0.5% topical timolol gel three times per day for 12 weeks	N/A	5/5
Meseguer‐Yerbra et al.,^33^ 2015	Case report (*n* = 2)	86 (78–94)	1/1	All negative	2 CKS	None	All PR	0.5% topical timolol gel two times per day for 12 weeks	No recurrence of KS after 20 and 22 months follow‐up	5/4
Sainz‐Gaspar et al.,^34^ 2017	Case report (*n* = 5)	71	1/0	Negative	CKS	None	CR	0.5% topical timolol gel two times per day for 24 weeks	No recurrence of KS after 10 months follow‐up	5/5

Abbreviations: AIDS‐KS, AIDS‐related KS; CKS, Classical KS; CR, Complete Response; IKS, Iatrogenic KS; LOE, level of evidence; NOS, Newcastle‐Ottawa Quality Assessment Scale; NR, no response; PD, progressive disease; PR, partial response; SD, stable disease.

^a^ Level of evidence rated using modified Oxford Centre for evidence‐based medicine.

^b^ The risk of bias for included studies assessed using Newcastle‐Ottawa Quality Assessment Scale.

**TABLE 4:** Summary of demographics, clinical characteristics and outcomes of other topical agents for cutaneous Kaposi sarcoma (KS)

Source, y	Study design	Age, median (range)	Male/female No.	HIV status	Type of KS	Known presence of extracutaneous diseases	Response	Regimen	Sustained response	LOE^a^/NOS score^b^
Pagliarello et al.,^36^ 2017	Case report (*n* = 2)	75 (70–80)	1/1	All negative	2 CKS	None	All CR	0.2% DPCP solution once weekly under occlusion for 24 h	No recurrence of KS after 4 and 6 months follow‐up	5/7
Goedert et al.,^35^ 2008	Randomized phase II clinical trial (*n* = 24)	67.5 (57–77)	19/5	All negative	24 CKS	None	(*n* = 24) 3 Missing data, 2 CR, 4 PR, 0 MR, 8 NR, 7 PD	7 mg 1/4‐size nicotine patches were applied for weeks 1–2, then 1/2‐size patches for 2 weeks, and full‐size patches for weeks 5–15	Did not follow‐up	1/7
Bonhomme et al.,^37^ 1991	Case series (*n* = 8)	35.5 (20–51)	8/0	All HIV+	8 AIDS‐KS	None	7 PR, 1 CR	1% tretinoin gel daily for 3 months	Did not follow‐up	4/5
Bonnetblanc et al.,^38^ 1994	Case report (*n* = 2)	32 (30–34)	2/0	All HIV+	2 AIDS‐KS	None	1 CR, 1 PR	BLM diluted in DMSO applied daily for 3 days for a total of five to six courses	Both cases had recurrence of the KS lesions after about a year	5/5
de Socarraz et al.,^49^ 1993	Case series (*n* = 8)	25–43	8/0	All HIV+	8 AIDS‐KS	None	4 PR	90% DMSO daily two to three times a day for 8 weeks	Progression of the lesions after 2 weeks follow‐up	4/4
Díaz‐Ley et al.,^39^ 2015	Case report (*n* = 1)	73	1/0	Negative	CKS	None	CR	0.5% rapamycin ointment every 12 h for 16 weeks.	No recurrence of KS after 24 months follow‐up	5/7
Cohen et al.,^40^ 1979	Case report (*n* = 4)	69.5 (39–70)	4/0	Not tested	4 CKS	1 case ‐ feet oedema, 1 case ‐ feet and left leg swelling,	All CR	BCG and cord factor ointment applied at every 1–3 weeks for up to 1 year	No recurrence of KS after 6 and 12 months follow up. 2 did not follow up.	5/7
Eilender et al.,^9^ 2006	Multicenter study (*n* = 4)	64—mean (30–86)	–	Not tested	Did not specify	One case—intestinal lesions bleeding	2 PR, 2 NR	0.25% A–007 gel two times a day for 3, 10, 16 and 17 weeks	Did not follow‐up	2/7
Masood et al.,^10^ 2000	Case series (*n* = 8)	43 (29–52)	‐	7 pts HIV+	7 AIDS‐KS, 1 CKS	None	1 CR, 3 PR, 4 NR	0.005% calcipotriene ointment two times per day for 3, 4, 10, 13, 24 months	Did not follow‐up	4/5
Koon et al.,^41^ 2011	Phase II clinical trial (*n* = 17)	43.1 (23–62)	17/0	All HIV+	17 AIDS‐KS	None	6 PR	Halofuginone ointment two times per day for 12 weeks	Did not follow‐up	1/8

Abbreviations: AIDS‐KS, AIDS‐related KS; CKS, Classical KS; CR, Complete Response; IKS, Iatrogenic KS; LOE, level of evidence; NOS, Newcastle‐Ottawa Quality Assessment Scale; NR, no response; PD, progressive disease; PR, partial response; SD, stable disease.

^a^ Level of evidence rated using modified Oxford Centre for evidence‐based medicine.

^b^ The risk of bias for included studies assessed using Newcastle‐Ottawa Quality Assessment Scale.

### 5.2 Topical alitretinoin for KS treatment

Three case reports and three clinical trials reported topical alitretinoin (9‐*cis*‐retinoic acid) for the topical treatment of cutaneous KS.^12^, ^13^, ^14^, ^15^, ^16^, ^17^ Five studies reported using 0.1% topical alitretinoin gel as the dosage^12^, ^14^, ^15^, ^16^, ^17^ and in one clinical trial, the concentration of topical alitretinoin ranged from 0.05% to 0.1%.^13^ The duration of the treatment regimen ranged from 8 to 78 weeks with a median of 12 weeks. There were varying drug frequencies, with twice daily being the most common regimen (Table 1).

Of three patients in the case reports, two achieved complete clinical response and one had no response. From a total 194 patients across two clinical trials using topical alitretinoin for cutaneous KS, 2 reported complete response (1.0%), 68 reported partial response (35.1%), 94 reported stable disease (48.5%), and 32 reported progressive disease (16.5%).^12^, ^17^ Both trials reported a statistically significant difference (*p* = 0.00003^12^ and *p* = 0.002^17^) in outcomes between topical alitretinoin and the control group. In the third clinical trial of 115 patients, there was a statistically significant difference in treatment response between topical alitretinoin and the untreated control group (*p* < 0.001).^13^ However, the level of response achieved in the treatment group was not clearly delineated.^13^

One study reported no recurrence of cutaneous KS after 1.5 years follow up^15^ and another study reported remission of 90%–100% of the lesions after 3–5 months^16^ Four studies did not follow up for the examination of cutaneous KS recurrence (Table 1).^12^, ^13^, ^14^, ^17^ In all six studies, of 314 patients treated with topical alitretinoin, 212 patients (67.5%) experienced rash, 50 patients (15.9%) experienced skin disorders and 46 patients (14.6%) experienced pruritus as the most common side effects. The nature of the rash and skin disorder was not clearly defined.

### 5.3 Topical imiquimod for KS treatment

Eight case reports, one prospective phase II cohort study and one comparative single‐blinded noncontrolled clinical study utilized topical imiquimod as a treatment for cutaneous KS.^11^, ^18^, ^19^, ^20^, ^21^, ^22^, ^23^, ^24^, ^25^, ^26^ All studies used 5% imiquimod cream, and the duration of treatment ranged from 6 to 83 weeks, with a median of 17 weeks (Table 2). Six studies applied topical imiquimod under occlusion ranging from 8 to 10 h,^11^, ^19^, ^21^, ^23^, ^24^, ^25^ and four studies reported application without occlusion.^18^, ^20^, ^22^, ^26^ Overall, in the 26 patients treated with topical imiquimod, 9 patients (34.6%) achieved complete clinical response, 8 patients (30.8%) achieved partial response and no data were reported for the remaining 9 patients (34.6%). In one study, of the 26 KS lesions in 8 patients treated with topical imiquimod, 11 lesions achieved complete clinical response (43.3%), 8 lesions partial response (30.8%), 4 lesions fair response (15.4%) and 3 lesions no response (11.5%).^26^ In this study, all lesion scores (depth, surface and total number) showed a statistically significant decrease at the end of 12 weeks using topical imiquimod (*p* = 0.001).^26^ Eight studies reported no recurrence of cutaneous KS lesions after a median follow up of 12 months (range: 6–15 months [Table 2]).^18^, ^19^, ^20^, ^21^, ^22^, ^23^, ^24^, ^25^ One study reported tumour progression in six patients^11^ and another did not follow up for KS recurrence.^26^ Of the 34 patients treated with topical imiquimod, 13 (38.2%) reported local pruritus and erythema at the site of application and 2 (6.0%) reported flu‐like symptoms after application.

### 5.4 Topical timolol for KS treatment

Eight case reports/series documented the use of topical timolol as a treatment for cutaneous KS.^27^, ^28^, ^29^, ^30^, ^31^, ^32^, ^33^, ^34^ One study did not specify the regimen,^30^ but six studies reported using 0.5% topical timolol^28^, ^29^, ^31^, ^32^, ^33^, ^34^ whereas one study used 0.1% topical timolol.^27^ Treatment duration ranged from 4 to 24 weeks, with a median of 12 weeks. There were varying medication frequencies, with twice daily application the most common regimen (Table 3). Of 13 patients treated with topical timolol, eight patients (61.5%) had complete clinical response, four patients (30.8%) had partial response and one patient (7.7%) had no response. Seven studies reported no recurrence of KS after variable months of follow up with a median of 12 weeks and a range of 4–24 weeks (Table 3). There were no treatment‐related side effects associated with the use of topical timolol. One patient reported asthenia and fever; however, the authors attributed these symptoms to HIV and active tuberculosis during treatment.^27^

### 5.5 Other topical treatments for KS treatment

Nicotine patch was used as a treatment modality in one clinical trial, and of 24 patients using nicotine patch, 2 achieved complete response (8.3%), 4 achieved partial response (16.7%), none achieved minor response (0%), 8 had no response (33.3%), and 7 had disease progression (29.2%).^35^ In this study, there was no statistically significant response between nicotine patch and the untreated control lesions (*p* = 0.74).^35^ Topical diphencyprone (DPCP) in 0.2% gel was used in a case report with two patients; both achieved complete response.^36^ One case series used all‐trans‐retinoic acid as a topical treatment for KS in eight patients.^37^ In this case series, one patient achieved complete response and seven achieved partial response.^37^ Another case series used topical bleomycin‐dimethylsulfoxide as topical KS treatment in eight patients, with all achieving partial response and the disappearance of pain.^38^ Topical 0.5% rapamycin ointment provided complete response for one patient in a case study.^39^ Topical BCG and cord factor was used for four patients in a case study and all achieved complete response.^40^ In one multicenter study, 4,4′‐dihydroxybenzophenone‐2,4‐dinitrophenylhydrazone (A007) was used a topical KS treatment in four patients; two achieved partial response and two had no response.^9^ Topical calcipotriene was used in eight patients in a single case series; of eight patients, one achieved complete response, three achieved partial response and four had no response.^10^ In one clinical trial of 17 patients using topical halofuginone as KS treatment, 6 patients achieved partial response with no statistical difference between the treatment and the control groups (*p* = 0.689).^41^ For detailed clinical characteristics and outcomes of less widely used topical agents for cutaneous KS see Table 4.

## 6 DISCUSSION

This systematic review aimed to identify topical treatments for cutaneous KS and evaluate the clinical efficacy of each treatment modality. We also investigated the most widely used treatment regimens and dosages, along with the drug‐related adverse effects. Based on our review, the three most common topical treatments were timolol, imiquimod and alitretinoin. These three drugs were easily administered topical medications with minimal drug‐related adverse events.

Alitretinoin is the only FDA‐approved topical medication for KS, with a labelled indication for AIDS‐related cutaneous KS. As with other retinoids, alitretinoin binds to and activates intracellular retinoid receptors; this results in altered gene expression that affects cellular differentiation and proliferation.^13^

Imiquimod is an immune response modifier that activates immune cells via Toll‐like receptor seven agonist activity. It is FDA‐approved for treatment for actinic keratoses, superficial basal cell carcinoma, and genital/perineal warts. However, it has been used for many off‐label indications, including the treatment of cutaneous KS. Imiquimod's anti‐angiogenic properties are thought to be mediated by cytokine induction (including IL‐10 and IL‐12), local upregulation of endogenous angiogenesis inhibitors (including TIMP, TSP‐1), local downregulation of pro‐angiogenic factors (including bFGF, MMP9), and promotion of endothelial cell apoptosis.^11^

Timolol is a nonselective beta‐blocker; as a topical ophthalmic agent it has a labelled indication for treatment of elevated intraocular pressure. The topical gel‐forming solution also has demonstrable efficacy for treatment of superficial infantile hemangiomas, and has been widely used by dermatologists for this purpose.^42^ The mechanism by which beta blockade mitigates vascular proliferation is not completely understood, but is hypothesized to be mediated by vasoconstriction, inhibition of angiogenesis and induction of apoptosis.^28^ Given that KS is a vascular tumour, it is not surprising that lesions would respond to topical timolol treatment. In addition, there are data to suggest that KS lesions may strongly express beta adrenergic receptors,^43^ and therefore may be particularly susceptible to beta‐blocker treatment. While the use of topical timolol yielded good response rates in our review, it should be noted that this evidence is based exclusively on case reports and case series. Stronger level of evidence, such as randomized controlled trials, are needed to fully evaluate the efficacy of this drug.

Our review also identified reports of less widely used topical treatments for cutaneous KS. These included topical diphencyprone (DPCP), all‐*trans*‐retinoic‐acid, rapamycin and bleomycin‐dimethylsulfoxide (BLM‐DMSO) which achieved variable response rates but have not been widely studied.

The demographics of KS patients in our review are consistent with the published epidemiological data of this disease.^44^ The majority of patients with CKS in our included studies was elderly males with a median age of 75 years, while AIDS‐related KS was prevalent in HIV positive younger males with a median age of 38.5 years. Overall, our reviewed studies included a significantly higher number of male patients than female patients. Extracutaneous KS may involve the viscera and lymph nodes; it is more difficult to treat and has a poorer prognosis than exclusive cutaneous disease.^45^ In most studies in our review, clinical outcome was not stratified based on the extracutaneous KS involvement.

The data on treatment response stratified by lesion size was inconclusive. One study reported a statistically significant positive correlation between small initial tumour area at the beginning of treatment and the area response at the end of treatment (*p* = 0.0246).^11^ Another study did not find any correlation between initial size of the lesions and response rate.^26^

### 6.1 Limitations

Our systematic review was limited by the inclusion of very few RCTs. Secondly, the heterogeneity of studies in describing clinical outcomes made it difficult to provide summary statistics for each topical agent. We found that some studies used AIDS Clinical Trials Group Oncology Committee criteria while others used the modified KS staging system to define clinical response.^46^, ^47^ A few studies did not clearly define the criteria for determining clinical response, which could be a source of investigator bias. Additionally, some patients in our included studies were on concurrent medications and had undergone prior KS therapies such as cryotherapy, local radiation and excisional surgery. This could potentially confound clinical results and introduce bias in reporting clinical outcomes. In some studies, immunosuppressive medications were tapered at the time of KS diagnosis, which may also affect the validity of results. In addition, variable rates of follow‐up to assess for cutaneous KS recurrence after topical therapy limited our ability to comment on sustained treatment efficacy.^48^

## 7 CONCLUSION

Alitretinoin, imiquimod and timolol were the three most common topical agents used to treat cutaneous KS. All three showed clinical efficacy with minimal drug‐related adverse events. There were also reports of less widely used topical treatments which showed variable clinical efficacy for cutaneous KS. However, further randomized controlled trials are needed to more accurately determine clinical outcomes, regimens and adverse effects for these agents. In the absence of standardized clinical guidelines on cutaneous KS therapy, our study provided a comprehensive list of topical treatments, and a summary of the available data on their efficacy, to better inform clinicians regarding treatment decisions.

## CONFLICT OF INTEREST

All of the authors have no conflict of interest.

## ETHICAL APPROVAL

Not required.

## AUTHOR CONTRIBUTIONS

**Kyaw Zin Htet:** Conceptualization‐Lead, Data curation‐Lead, Formal analysis‐Lead, Investigation‐Equal, Methodology‐Lead, Project administration‐Equal, Writing – original draft‐Equal, Writing – review & editing‐Equal. **Michael Andrew Waul:** Conceptualization‐Equal, Data curation‐Equal, Formal analysis‐Equal, Investigation‐Equal, Methodology‐Equal, Project administration‐Equal, Supervision‐Equal, Writing – original draft‐Equal, Writing – review & editing‐Equal. **Kieron Seymour Leslie:** Conceptualization‐Lead, Data curation‐Equal, Investigation‐Equal, Project administration‐Equal, Supervision‐Lead, Writing – original draft‐Supporting, Writing – review & editing‐Supporting.

## Supporting information

Supplementary Material

## Data Availability

Data sharing is not applicable to this article as no new data were created or analyzed in this study.
