# Supplementary material for: Topical treatments for Kaposi sarcoma: A systematic review
Source: Skin Health Dis. 2022 Apr 8;2(2):e107. doi: 10.1002/ski2.107 (PMC9168015; doi:10.1002/ski2.107)
Supplement: Supplementary file 1 — Supplementary Material [file SKI2-2-e107-s001.docx]

**Search Strategy -**

**Embase total results:**

('kaposi sarcoma'/exp OR 'kaposi sarcoma' OR 'kaposis sarcoma' OR 'sarcoma, kaposi') AND ('topical treatment'/exp OR 'topical drug administration, therapeutic' OR 'topical therapy' OR 'topical treatment' OR 'treatment, topical' OR 'topical drug administration'/exp OR 'administration, topical' OR 'drug administration, topical' OR 'topic administration' OR 'topic application' OR 'topic medication' OR 'topic therapy' OR 'topic treatment' OR 'topical administration' OR 'topical application' OR 'topical drug administration') AND ('remission'/exp OR 'disease regression' OR 'disease remission' OR 'regression, disease' OR 'remission' OR 'remission induction' OR 'remission rate' OR 'remission, spontaneous' OR 'spontaneous regression' OR 'cancer regression'/exp OR 'cancer regression' OR 'cancer remission' OR 'regression, cancer' OR 'spontaneous cancer regression' OR 'spontaneous cancer remission' OR 'drug efficacy'/exp)

**Pubmed:**

((("sarcoma, kaposi"[MeSH Terms] OR ("sarcoma"[All Fields] AND "kaposi"[All Fields])) OR "kaposi sarcoma"[All Fields]) OR ("kaposi"[All Fields] AND "sarcoma"[All Fields])) AND (((((("neoplasm regression, spontaneous"[MeSH Terms] OR (("neoplasm"[All Fields] AND "regression"[All Fields]) AND "spontaneous"[All Fields])) OR "spontaneous neoplasm regression"[All Fields]) OR (("neoplasm"[All Fields] AND "regression"[All Fields]) AND "spontaneous"[All Fields])) OR "neoplasm regression spontaneous"[All Fields]) OR ((((remission) OR (regression)) AND (cancer)) OR (neoplasm))) AND (((("administration, topical"[MeSH Terms] OR ("administration"[All Fields] AND "topical"[All Fields])) OR "topical administration"[All Fields]) OR ("topical"[All Fields] AND "administration"[All Fields])) OR ((("topical"[All Fields] OR "topically"[All Fields]) OR "topicals"[All Fields]) AND (((((("therapeutics"[MeSH Terms] OR "therapeutics"[All Fields]) OR "treatments"[All Fields]) OR "therapy"[MeSH Subheading]) OR "therapy"[All Fields]) OR "treatment"[All Fields]) OR "treatment s"[All Fields]))))

Table 5. Risk of Bias Assessment for Included Cohort Studies, Case Control Studies and Randomized Controlled Trials based on Newcastle-Ottawa Quality Assessment Scale

| Author(s), Year | Study Design | Selection^a^ | Comparability^b^ | Exposure/Outcome^c^ | Total NOS score^d^ |
| --- | --- | --- | --- | --- | --- |
| Célestin Schartz et al,^10^ 2008 | Prospective Phase II Cohort Study | 2 | 0 | 2 | 4 |
| Odyakmaz Demirsoy et al,^25^ 2019 | Comparative single-blinded noncontrolled clinical study | 2 | 0 | 2 | 4 |
| Bodsworth et al,^11^ 2001 | Randomized phase III double-blinded study | 3 | 2 | 3 | 8 |
| Duvic et al,^12^ 2000 | Randomized phase I and II open-label controlled study | 3 | 1 | 3 | 7 |
| Walmsley et al,^16^ 1999 | Randomized double-blinded multi-center clinical trial | 3 | 2 | 3 | 8 |
| Goedert et al,^34^ 2008 | Randomized phase II clinical trial | 3 | 1 | 3 | 7 |
| Eilender et al,^8^ 2006 | Multicenter Study | 3 | 1 | 3 | 7 |
| Koon et al,^40^ 2011 | Phase II Clinical Trial | 3 | 2 | 3 | 8 |

^a^ Maximum of 4 points

^b^ Maximum of 2 points

^c^ Maximum of 3 points

^d^ Maximum of 9 total points

Table 6. Risk of Bias Assessment for Included Case Reports and Case Series based on Modified Newcastle-Ottawa Quality Assessment Scale

| Author(s), Year | Study Design | Selection^a^ | Ascertainment^b^ | Causality^c^ | Reporting^d^ | Total NOS score^e^ |
| --- | --- | --- | --- | --- | --- | --- |
| Abdelmaksoud et al,^26^ 2017 | Case Series | 1 | 2 | 3 | 1 | 7 |
| Alcántara-Reifs et al,^27^ 2016 | Case Report | 1 | 2 | 1 | 1 | 5 |
| Chap et al,^28^ 2017 | Case Report | 1 | 2 | 2 | 1 | 6 |
| Deutsch et al,^29^ 2018 | Case Report | 1 | 1 | 1 | 0 | 3 |
| Espadafor-López et al,^30^ 2020 | Case Report | 1 | 2 | 2 | 1 | 6 |
| Gupta et al,^31^ 2019 | Case Report | 1 | 2 | 1 | 1 | 5 |
| Meseguer-Yerbra et al,^32^ 2015 | Case Report | 1 | 1 | 1 | 1 | 4 |
| Sainz-Gaspar et al,^33^ 2017 | Case Report | 1 | 2 | 1 | 1 | 5 |
| Babel et al,^17^ 2007 | Case Report | 1 | 2 | 3 | 1 | 7 |
| Benomar et al,^18^ 2009 | Case Report | 1 | 2 | 1 | 1 | 5 |
| Fairley et al,^19^ 2012 | Case Report | 1 | 2 | 2 | 1 | 6 |
| Goiriz et al,^20^ 2008 | Case Report | 1 | 2 | 1 | 1 | 5 |
| Rosen,^21^ 2006 | Case Report | 1 | 2 | 3 | 1 | 7 |
| Bernardini et at,^22^ 2010 | Case Report | 1 | 2 | 2 | 1 | 6 |
| Gündüz et al,^23^ 2012 | Case Report | 1 | 2 | 1 | 1 | 5 |
| Prinz Vavricka et al,^24^ 2011 | Case Report | 1 | 2 | 4 | 1 | 8 |
| González de Arriba et al,^13^ 2007 | Case Report | 1 | 2 | 2 | 1 | 6 |
| Morganroth,^14^ 2002 | Case Report | 1 | 2 | 3 | 1 | 7 |
| Rongioletti et al,^15^ 2006 | Case Report | 1 | 2 | 3 | 1 | 7 |
| Pagliarello et al,^35^ 2017 | Case Report | 1 | 2 | 3 | 1 | 7 |
| Bonhomme et al,^36^ 1991 | Case Series | 1 | 2 | 1 | 1 | 5 |
| Bonnetblanc et al,^37^ 1994 | Case Report | 1 | 1 | 2 | 1 | 5 |
| de Socarraz et al,^47^ 1993 | Case Series | 1 | 1 | 1 | 1 | 4 |
| Díaz-Ley et al,^38^ 2015 | Case Report | 1 | 2 | 3 | 1 | 7 |
| Cohen et al,^39^ 1979 | Case Report | 1 | 2 | 3 | 1 | 7 |
| Masood et al,^9^ 2000 | Case Series | 1 | 2 | 1 | 1 | 5 |

^a^ Maximum of 1 point

^b^ Maximum of 2 points

^c^ Maximum of 4 points

^d^ Maximum of 1 point

^e^ Maximum of 8 total points
